# Function of glutathione peroxidases in legume root nodules

**DOI:** 10.1093/jxb/erv066

**Published:** 2015-03-04

**Authors:** Manuel A. Matamoros, Ana Saiz, Maria Peñuelas, Pilar Bustos-Sanmamed, Jose M. Mulet, Maria V. Barja, Nicolas Rouhier, Marten Moore, Euan K. James, Karl-Josef Dietz, Manuel Becana

**Affiliations:** ^1^Departamento de Nutrición Vegetal, Estación Experimental de Aula Dei, Consejo Superior de Investigaciones Científicas (CSIC), Apartado 13034, 50080 Zaragoza, Spain; ^2^Institut des Sciences du Végétal, Avenue de la Terrasse, 91198 Gif-sur-Yvette, France; ^3^Instituto de Biología Molecular y Celular de Plantas, Universidad Politécnica de Valencia-CSIC, Camino de Vera, 46022 Valencia, Spain; ^4^Université de Lorraine, Interactions Arbres-Microorganismes, UMR1136, F-54500 Vandoeuvre-lès-Nancy, France; ^5^INRA, Interactions Arbres-Microorganismes, UMR1136, F-54280 Champenoux, France; ^6^Biochemistry and Physiology of Plants, W5-134, Bielefeld University, D-33501 Bielefeld, Germany; ^7^The James Hutton Institute, Invergowrie, Dundee DD2 5DA, UK

**Keywords:** Antioxidants, glutathione peroxidases, legume nodules, *Lotus japonicus*, nitric oxide, reactive oxygen species, S-nitrosylation.

## Abstract

Glutathione peroxidases (Gpxs) are antioxidant enzymes not studied so far in legume nodules, despite the fact that reactive oxygen species are produced at different steps of the symbiosis. The function of two Gpxs that are highly expressed in nodules of the model legume *Lotus japonicus* was examined. Gene expression analysis, enzymatic and nitrosylation assays, yeast cell complementation, *in situ* mRNA hybridization, immunoelectron microscopy, and LjGpx-green fluorescent protein (GFP) fusions were used to characterize the enzymes and to localize each transcript and isoform in nodules. The *LjGpx1* and *LjGpx3* genes encode thioredoxin-dependent phospholipid hydroperoxidases and are differentially regulated in response to nitric oxide (NO) and hormones. LjGpx1 and LjGpx3 are nitrosylated *in vitro* or in plants treated with *S*-nitrosoglutathione (GSNO). Consistent with the modification of the peroxidatic cysteine of LjGpx3, *in vitro* assays demonstrated that this modification results in enzyme inhibition. The enzymes are highly expressed in the infected zone, but the LjGpx3 mRNA is also detected in the cortex and vascular bundles. LjGpx1 is localized to the plastids and nuclei, and LjGpx3 to the cytosol and endoplasmic reticulum. Based on yeast complementation experiments, both enzymes protect against oxidative stress, salt stress, and membrane damage. It is concluded that both LjGpxs perform major antioxidative functions in nodules, preventing lipid peroxidation and other oxidative processes at different subcellular sites of vascular and infected cells. The enzymes are probably involved in hormone and NO signalling, and may be regulated through nitrosylation of the peroxidatic cysteine essential for catalytic function.

## Highlight

Glutathione peroxidases are antioxidant enzymes localized to different cell compartments, including the nucleus. Transcriptional and post-translational regulation via S-nitrosylation strongly suggest functions in hormonal cascades and nitric oxide redox signalling.

## Introduction

Glutathione peroxidases (Gpxs) are ubiquitous enzymes that catalyse the reduction of H_2_O_2_ or organic peroxides to water or the corresponding alcohols using glutathione (GSH) or thioredoxins (Trxs) as electron donors (Herbette *et al.*, 2007; Brigelius-Flohé and Maiorino, 2013). These enzymes were initially described in mammals, where eight clades can be distinguished based on amino acid sequences, substrate specificity, and subcellular localization (Herbette *et al.*, 2007; Brigelius-Flohé and Maiorino, 2013). Four groups of Gpxs, termed ‘classical’ or cytosolic (Gpx1), gastrointestinal (Gpx2), plasmatic (Gpx3), and phospholipid hydroperoxidases (Gpx4), contain seleno-Cys instead of Cys at the catalytic site. Gpx6, located in the olfactory system, is a selenoprotein in humans and pigs but not in rodents, whereas an epididymis-specific (Gpx5) and two recently discovered Gpxs associated to the endoplasmic reticulum (Gpx7 and Gpx8) do not contain seleno-Cys (Brigelius-Flohé and Maiorino, 2013).

Plant Gpxs are most similar in terms of amino acid sequences to the mammalian Gpx4 enzymes but lack seleno-Cys (Herbette *et al.*, 2007), with the single exception of the Gpx from the unicellular green alga *Chlamydomonas reinhardtii* (Fu *et al.*, 2002). The fact that Cys is less reactive than seleno-Cys may explain why plant Gpxs are less efficient in scavenging reactive oxygen species (ROS) than their mammalian counterparts (Herbette *et al.*, 2007). Plant Gpxs usually have three Cys residues (Supplementary Fig. S1), but only the first (‘peroxidatic’) Cys and the third (‘resolving’) Cys are required for catalysis and Trx-mediated regeneration (Navrot *et al.*, 2006; Koh *et al.*, 2007). The Gpxs are encoded by small multigene families, comprising five to eight members in the model plants so far examined (Rodriguez Milla *et al.,* 2003; Navrot *et al.*, 2006; Margis *et al.*, 2008; Ramos *et al.*, 2009). Many plant Gpxs may protect membranes from peroxidative damage (Gueta-Dahan *et al.*, 1997; Herbette *et al.*, 2002) and some *Arabidopsis thaliana* Gpx isoforms may play additional roles in redox transduction and stress signalling (Miao *et al.*, 2006; Chang *et al.*, 2009).

Legumes establish symbiotic associations with rhizobia forming root nodules, which are unique organs that fix atmospheric N_2_ into ammonium. Nodules contain O_2_-sensitive metalloproteins and leghemoglobin that favour ROS production (Dalton, 1995; Becana *et al.*, 2010). However, low steady-state ROS levels are required for critical functions such as plant organ development and stress perception (Foyer and Noctor, 2005; Puppo *et al.*, 2005). To offset the potential toxicity of ROS while allowing them to play signalling roles, nodules contain an impressive array of antioxidants, although only the enzymes and metabolites of the ascorbate-GSH pathway have been studied in detail to elucidate their role in peroxide metabolism (Dalton, 1995; Becana *et al.*, 2010). In sharp contrast, the function of Gpxs in nodules has been overlooked, despite early studies showing that Gpx activity is responsive to oxidative stress (Gueta-Dahan *et al.*, 1997) and that ROS and nitric oxide (NO) are involved at different stages of the symbiosis (Puppo *et al.*, 2013). Six *Gpx* genes have been identified in the model legume *L. japonicus* and two of them, *LjGpx1* and *LjGpx3*, are highly expressed in nodules (Ramos *et al.*, 2009). Here, a detailed characterization of these two isoforms is provided by combining enzyme activity assays, expression profiles, mRNA and protein localizations in nodules, and functional complementation of a yeast Gpx-deficient mutant. Because Gpx activities rely on critical Cys residues (Jung *et al.*, 2002; Navrot *et al.*, 2006; Herbette *et al.*, 2007) and *S*-nitrosylation is an important post-translational modification underlying NO signalling (Astier *et al.*, 2012), the possible regulation of LjGpx1 and LjGpx3 activities by nitrosylation has been studied by using dedicated mass spectrometry (MS) methods.

## Materials and methods

### Plant growth and treatments

Seeds of *Lotus japonicus* (Regel) Larsen ecotype MG20 were sown, seedlings were inoculated with *Mesorhizobium loti* strain R7A, and plants were grown in controlled environment cabinets as previously described (Ramos *et al.*, 2009). Plants used for biochemical and microscopy studies were grown for 46 d in pots (1 litre) containing vermiculite and were irrigated twice a week with B&D nutrient solution (Broughton & Dilworth, 1971) supplemented with 0.25mM NH_4_NO_3_.

Expression profiles of *LjGpx1* and *LjGpx3* were determined in nodules of plants exposed to stress and hormones. (i) *Nitro-oxidative stress.* This was induced by treating the plants with cadmium (Cd) or *S*-nitrosoglutathione (GSNO). Plants grown for 46 d in pots were separated into two groups. One set of plants was treated with 100 μM CdCl_2_ in water and nodules were harvested after 6h. The other set of plants was transferred to Erlenmeyer flasks containing 250ml of 1:10 HEN buffer [100mM HEPES (pH 8.0), 1mM EDTA, 0.1mM neocuproine] supplemented with either 5mM GSNO or glutathione disulfide (GSSG; control). The flasks were protected from light and plants were treated for 6h. (ii) *Phytohormones.* Nodulated plants were grown hydroponically for 44 d (Tovar-Méndez *et al.*, 2011) and treated for 48h with 50 μM abscisic acid (ABA), salicylic acid (SA), jasmonic acid (JA), 1-aminocyclopropane-1-carboxylic acid (ACC), or cytokinin (CK, an equimolar mixture of kinetin and 6-benzyl-aminopurine). Stock solutions (100mM) were prepared in 2ml of ethanol (ABA, ACC, SA), dimethylsulfoxide (JA), or 1M NaOH (CKs), and added to 4 l of the aerated hydroponic solution (1:4 B&D nutrient solution lacking combined nitrogen, pH 6.6). Control plants were treated with the same concentrations of ethanol, dimethylsulfoxide, or NaOH.

### Expression analysis of LjGpx genes

Total RNA was extracted from nodules and processed as described (Ramos *et al.*, 2009). Quantitative reverse-transcription PCR was performed with the primers listed in Supplementary Table S1 using a 7500 Real-Time PCR System (Applied Biosystems). Transcript levels were normalized with *ubiquitin* and the relative values of gene expression were calculated using the 2exp(-ΔΔ*C*_T_) method, where *C*_T_ is the threshold cycle (Livak and Schmittgen, 2001). The stability of *ubiquitin* expression during the treatments was confirmed with *eIF-4A* (eukaryotic initiation factor 4A) and *PP2A* (subunit of the Ser/Thr protein phosphatase 2A) as additional reference genes.

### Biochemical characterization of LjGpxs

#### Expression and purification of recombinant proteins

Fragments of *LjGpx1* and *LjGpx3* encoding the predicted mature proteins (Supplementary Fig. S1) were amplified by PCR from nodule cDNA using PfuUltra II DNA polymerase (Agilent) and primers (Supplementary Table S1) compatible with pET200 directional TOPO expression kits (Invitrogen). Protein expression was induced in *Escherichia coli* BL21 (DE3) by the addition of 1mM isopropyl-β-d-thiogalactopyranoside for 4h at 37ºC. Bacteria were harvested by centrifugation, resuspended in 50mM potassium phosphate (pH 8.0) containing 300mM NaCl and 40mM imidazole, and sonicated 6×30 s. Extracts were cleared by centrifugation and supernatants were loaded onto HiTrap chelating HP Ni-affinity columns (GE Healthcare Life Sciences). The His-tagged proteins were eluted with buffer supplemented with 250mM imidazole, desalted, and concentrated by ultrafiltration.

#### Biochemical assays

LjGpx1 and LjGpx3 activities were determined by monitoring NADPH oxidation at 340nm (extinction coefficient = 6.22mM^-1^ cm^-1^) under steady-state conditions. The reaction mixture comprised TE buffer [30mM Tris-HCl (pH 8.0), 1mM EDTA], 1 μM *A. thaliana* NADPH-Trx reductase, 20 μM poplar *(Populus trichocarpa)* Trx*h1*, 150nM recombinant enzymes, and 0.4mM NADPH (Navrot *et al.*, 2006). The activities were recorded using 0.5–30 μM phosphatidylcholine hydroperoxide and 5–1000 μM H_2_O_2_, *t*-butyl hydroperoxide, and cumene hydroperoxide. Phosphatidylcholine hydroperoxide was synthesized as described by Maiorino *et al.* (1990) and its concentration standardized by the FOX colorimetric method (Wolff, 1994). The Gpx activity was determined after subtracting the spontaneous reduction rate observed in the absence of Gpx. The apparent *K*_m_ and *V*_max_ values were calculated by nonlinear regression using a Michaelis-Menten equation. To study the effect of *S*-nitrosylation on enzyme activities, recombinant LjGpx1 and LjGpx3 were treated with 1mM GSNO or GSSG (control) for 1h at 37ºC in the dark. Excess reagents were removed by ultrafiltration and enzyme activity was assayed with H_2_O_2_ as described above.

#### Interaction of LjGpxs with endogenous Trxs

**Fig. 1.**
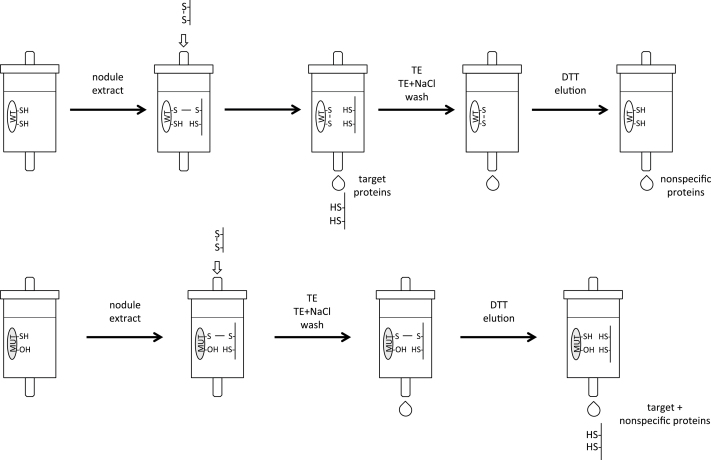
Procedure followed to demonstrate the interaction between the cytosolic thioredoxin LjTrx*h4* and LjGpxs. Two CNBr-Sepharose columns were prepared by covalently binding wild-type (WT) and mutated (MUT) LjTrx*h4*. These columns were loaded with identical protein amounts from soluble nodule extracts. After several washes with Tris-EDTA buffer (TE) without and with NaCl, the proteins interacting with each of the LjTrx*h4* proteins were eluted using a DTT-containing buffer. Nodule proteins retained by the mutated LjTrx*h4* but not by the wild-type LjTrx*h4* were considered as thioredoxin targets.

The procedure of Balmer *et al.* (2003) was followed as shown schematically in Fig. 1. The *L. japonicus* Trx*h4* (LjTrx*h4*) and a mutated derivative (Cys-60-Ser), produced by site-directed mutagenesis, were cloned using specific primers (Supplementary Table S1) as indicated for LjGpxs. Both proteins had an N-terminal poly-His tag and were purified by Ni-affinity chromatography. Purified Trxs (7mg) were bound to CNBr-activated Sepharose 4B (1.25g Sepharose) following the manufacturer’s instructions (GE Healthcare Life Sciences). Extracts of *L. japonicus* nodules were prepared in TE+protease inhibitor cocktail (Roche). The extracts were cleared by centrifugation and the supernatants were separated into two fractions, which were passed through the columns containing either the wild-type or the mutated proteins. The columns were previously washed with TE+2mM DTT to ensure complete reduction of bound Trxs, and then with TE alone to remove excess DTT. The nodule extracts (25–40mg of protein) were passed continuously overnight through the columns, which were afterwards washed with five volumes of TE buffer and another five volumes of TE+500mM NaCl. The bound proteins were then eluted with TE+10mM DTT and identified by liquid chromatography coupled to tandem mass spectrometry (LC-MS/MS) in both data-dependent and target acquisition modes. In the latter case, between four and nine tryptic peptides were searched for each LjGpx protein. The MS instrument was a Velos LTQ (Thermo Scientific) equipped with a microelectrospray ionization source. Samples containing 2 μg protein were diluted up to 20 μl with 5% methanol and 1% formic acid, and loaded on the chromatographic system. Details of the chromatography and detection conditions are given in Sainz *et al.* (2015).

### Complementation of LjGpxs in yeast

Methods for yeast (*Saccharomyces cerevisiae*) manipulation and for preparation of rich yeast extract-peptone-dextrose medium (YPD) and minimal synthetic-dextrose growth medium (SD) were as described by Guthrie and Fink (1991). YPD was used for experiments and SD for selecting transformant colonies and precultures. Complementation with LjGpx1 and LjGpx3 was carried out with the triple deletion mutant *gpx1Δ/2Δ/3Δ* (MATa *his3Δ1 leu2Δ0 met15ΔΔ0 ura3Δ0 gpx1::URA3 gpx2::His3MX6 gpx3::KanMX6*) derived from the BY4741 strain (Avery and Avery, 2001). The constructs encoding the mature LjGpx1 and LjGpx3 proteins were cloned into pENTR/D-TOPO and recombined into the yeast expression vector pAG425GPD-ccdB using Gateway LR Clonase II (Invitrogen). The mutant strain was transformed with the constructs by the lithium acetate-polyethylene glycol method (Gietz and Woods, 2002). Growth assays were performed in solid YPD medium by spotting serial dilutions of saturated cultures onto plates with the concentrations of stress inducers and the exposure times as indicated. The peroxides were added on top of the solidified medium, whereas NaCl and caffeine were added prior to autoclaving. Linolenic acid was prepared from a concentrated stock in YPD medium containing 1% (w/v) tergitol (Avery and Avery, 2001) and supplied to the medium after autoclaving but prior to gelification. Control experiments with media supplemented with 1% tergitol alone showed no effect on yeast growth.

### Localization of LjGpx transcripts and proteins in nodules

In situ *RNA hybridization* Antisense and sense digoxigenin-labelled RNA probes based on gene-specific primers (Supplementary Table S1) were synthesized using the DIG RNA Labeling Kit (Roche). The protocols of Bustos-Sanmamed *et al.* (2013) were followed and the process was fully automated with an InsituPro VSi instrument (Intavis, Germany). Nodule sections were examined with a DMI6000 B inverted microscope (Leica).

#### Immunoblots

Antisera were raised in rabbits with *~*1mg of purified recombinant LjGpx1 and LjGpx3 proteins and were used to purify polyclonal monospecific antibodies by chromatography in CNBr-activated Sepharose 4 Fast Flow following conventional protocols (BioGenes, Germany). The antibodies were further purified by immunoadsorption with protein extracts of *E. coli*. Immunoblots were performed as described (Rubio *et al.*, 2009). The secondary antibody was a goat anti-rabbit IgG horseradish peroxidase conjugate (Sigma). The primary and secondary antibodies were used at dilutions of 1:500 and 1:20 000, respectively, and immunoreactive proteins were detected by chemiluminescence.

#### Immunogold localization

Nodules were fixed in 4% paraformaldehyde and 0.1% glutaraldehyde in 50mM sodium cacodylate buffer (pH 7.0). Procedures for sample dehydration in ethanol and infiltration in LR White resin at low temperatures were performed in a Leica AFS2 as described (Rubio *et al.*, 2009; Sainz *et al.*, 2013). Ultrathin sections were collected on pyroxylin-coated Ni-grids and incubated for 1h with each antibody diluted 1:10 in blocking/diluting buffer. The sections were then washed and incubated for 1h with 15-nm gold particles conjugated to protein A (BB International, UK) diluted 1:100 in the same buffer (Rubio *et al.*, 2009). Serial sections treated with non-immune serum substituting for LjGpx antibodies served as negative controls. Sections were viewed and digitally photographed using a JEM 1400 transmission electron microscope (JEOL, Japan).

#### Localization using GFP fusions and protoplast transformation

The open reading frames of *LjGpx1* and *LjGpx3*, bearing the sequences encoding the putative transit peptides, were amplified by PCR, cloned into pENTR/D-TOPO (Invitrogen), and recombined into the Gateway binary vector pGWB5 (Nakagawa *et al.*, 2007) with LR Clonase II. In these constructs, the green fluorescent protein (GFP) was translationally fused at the C-terminus of the LjGpx proteins and the expression of the fusion protein was driven by the cauliflower mosaic virus 35S promoter. Mesophyll protoplasts were isolated from *A. thaliana* leaves and 5 μg plasmid DNA was delivered into protoplasts by the downsized polyethylene glycol-mediated transfection method (Seidel *et al.*, 2004). Subcellular localization was visualized with a confocal laser scanning microscope (LSM 780, Zeiss, Germany) using excitation at 488nm (GFP and chlorophyll) and emission at 499–535nm (GFP) and 650–700nm (chlorophyll).

### Detection of S-nitrosylation of LjGpx1 and LjGpx3

This was performed using the biotin (Jaffrey *et al.*, 2001) and His-tag (Camerini *et al.*, 2007) switch assays.

#### Biotin switch assay

Recombinant LjGpx1 and LjGpx3 were diluted to 1mg ml^-1^ in HEN buffer and incubated with 1mM GSNO or GSSG (control) for 1h at 37ºC in the dark with shaking. Reagents were removed by acetone precipitation and two washes with ice-cold acetone. Free thiols were blocked in HEN buffer with 100mM *N*-ethylmaleimide (NEM) and 2.5% SDS for 1h at 37ºC in the dark with shaking. Excess NEM was removed by acetone precipitation/washing and proteins were solubilized in HENS buffer (HEN+1% SDS). The biotin switch was performed for 1h at 37ºC in the dark in HENS buffer containing 20mM ascorbate and 0.25mg ml^-1^ HPDP-Biotin (Pierce). Excess reagents were removed by acetone precipitation and washing. Proteins were resuspended in HENS buffer, separated on 15% SDS gels, and transferred onto polyvinylidene fluoride membranes. Anti-biotin antibody (Sigma) was used at 1:10000.

#### His-tag switch assay

Incubation with GSNO and derivatization of free thiols with 100mM NEM were as described for the biotin switch but replacing biotin by the alkylating peptide I-CH_2_-CO-Gly-Arg-Ala-(His)_6_. After incubation for 1h at 37ºC in the dark, proteins were dialysed overnight in 10mM NH_4_HCO_3_, concentrated, and analysed by matrix-assisted laser desorption/ionization time-of-flight MS.

#### Affinity purification of biotinylated proteins

Nodulated plants were treated with 5mM GSNO or GSSG for 6h. Proteins were extracted in HEN buffer with 0.2% SDS and protease inhibitors, and subjected to the biotin switch. Dry pellets were resuspended in binding buffer consisting of 25mM HEPES (pH 7.7), 1mM EDTA, 100mM NaCl, 0.8% Triton X-100, and 50 μl of streptavidin-agarose resin (Sigma). Samples were incubated overnight at 4ºC and then the agarose beads were washed ten times with a buffer comprising 25mM HEPES (pH 7.7), 1mM EDTA, 600mM NaCl, and 0.8% Triton X-100. Biotinylated proteins were eluted by boiling the beads for 10min in SDS loading buffer [50mM Tris-HCl (pH 6.8), 10% glycerol, 1% SDS, 0.01% bromophenol blue, 50mM DTT]. After centrifugation, proteins were separated on 15% SDS gels and transferred to membranes for immunoblot analysis with LjGpx antibodies.

## Results

### LjGpx1 and LjGpx3 are Trx-dependent phospholipid hydroperoxidases

Previous work had shown that *LjGpx1* and *LjGpx3* are highly expressed in nodules (Ramos *et al.*, 2009) and that the *LjGpx3* mRNA level is 6.8-fold greater in nodules than in uninfected roots (Colebatch *et al.*, 2002). These observations prompted us to focus on the function of LjGpx1 and LjGpx3. To this end, recombinant enzymes were produced and their activities assayed toward various hydroperoxides using Trx and GSH as potential electron donors. However, no LjGpx activity was detected with GSH as reductant and with H_2_O_2_ or organic peroxides as substrates, and hence further work was done exclusively with Trx. Kinetic analyses indicated that the two LjGpx isoforms catalyse the Trx-dependent reduction of H_2_O_2_, *t*-butyl hydroperoxide, and cumene hydroperoxide (Table 1). The apparent affinities of both isoforms for organic peroxides (*K*_m_ ~60–300 μM) were lower than for H_2_O_2_ (*K*_m_ ~20 μM). The opposite trend was seen for the maximum velocities (*V*_max_), with apparent values of ~4 μmol min^-1^ mg^-1^ protein for H_2_O_2_ and 4–15 μmol min^-1^ mg^-1^ protein for organic peroxides. The apparent affinity of LjGpx1 and LjGpx3 for phospholipid hydroperoxides was much higher (*K*_m_ ~1.6 μM) and the apparent *V*_*max*_ of LjGpx3 doubled that of LjGpx1. As a result, the *V*_max_ */K*_m_ ratios of the two enzymes, which are an indication of their catalytic efficiencies, were very high for lipid peroxides (2–4.5), low for H_2_O_2_ (0.2), and very low for organic peroxides (0.02–0.07) (Table 1). All these data led us to conclude that LjGpx1 and LjGpx3 are Trx-dependent phospholipid hydroperoxidases.

**Table 1.** Kinetic parameters of LjGpxs with various hydroperoxides as substrates and poplar thioredoxin (Trxh1) as the electron donor

Enzyme	Peroxide	*V*_max_ (μmol min^-1^ mg^-1^)	*K*_m_ (μM)	*V*_max_/*K*_m_
LjGpx1	H_2_O_2_	3.8	15.6	0.24
	*t*-Butyl hydroperoxide	7.8	330.8	0.02
	Cumene hydroperoxide	4.8	64.9	0.07
	Phosphatidylcholine hydroperoxide	3.2	1.6	2.00
LjGpx3	H_2_O_2_	4.0	20.5	0.20
	*t*-Butyl hydroperoxide	3.6	166.6	0.02
	Cumene hydroperoxide	14.7	213.5	0.07
	Phosphatidylcholine hydroperoxide	7.2	1.6	4.50

The interaction between LjGpxs and endogenous Trxs was demonstrated using the cytosolic isoform LjTrx*h4* that is highly expressed in nodules (Tovar-Méndez *et al.*, 2011). The Cys-60-Ser derivative of LjTrx*h4* was used for an affinity binding assay based on the formation of a stable heterodisulfide bond between the remaining Cys of the active site and the Cys residues of the target proteins (Fig. 1; Balmer *et al.*, 2003). The wild-type protein served as a control for nonspecific binding. In three independent preparations of nodules, LjGpx1 and LjGpx3 were identified as protein targets because they become covalently bound to mutated LjTrx*h4* but not to the wild-type protein (Table 2), which indeed supports the Trx-dependency of the two LjGpx isoforms. LjGpx2 was also found to be a target of LjTrx*h4* (Table 2), but LjGpx4, LjGpx5, or LjGpx6 (Ramos *et al.*, 2009) were not detected even though the highly sensitive target mode was used in the MS analysis.

**Table 2.** Identification of LjGpxs that interact with LjTrxh4 Proteins from nodule extracts that interact with LjTrxh4 were trypsinized and the resulting peptides were analysed by LC-MS/MS using the DDAM (data-dependent acquisition mode) or the TM (target mode). The mass to charge ratio (m/z) of the fragmented peptide ions are indicated. Three independent experiments were conducted, each one corresponding to a different nodule sample. +, ++, +++, positive identifications in one, two, or three experiments.

Proteins and peptides	*m/z*	DDAM	TM
**LjGpx1**			
FKAEFPVFDKVDVNGDSAAPLYK	853.10	++	+++
AEFPVFDKVDVNGDSAAPLYK	761.38	++	+++
VDVNGDSAAPLYK	674.84	++	+++
GNDVNLGDYK	547.76	+++	+++
FLVDKEGNVVER	702.88	++	+++
**LjGpx2**			
FKSEFPIFDKIEVNGENSAPLYK	891.46	+	+++
SEFPIFDKIEVNGENSAPLYK	799.73	+	
IEVNGENSAPLYK	717.37	++	
GSDVDLSTYK	1084.52	++	+++
WGIFGDDIQWNFAK	848.90	+++	+++
FLVDKDGQVVDR	695.87	++	+++
**LjGpx3**			
SLYDFTVK	486.75	+	+++
ELNILYEK	511.28	+	+++

### LjGpx1 and LjGpx3 are differentially expressed in response to nitro-oxidative stress and hormones, and the proteins protect against oxidative damage

Because both LjGpx isoforms are very active in reducing phospholipid hydroperoxides to innocuous lipid alcohols, they may protect cells from oxidative damage. This hypothesis was tested by two experimental approaches.

**Fig. 2.**
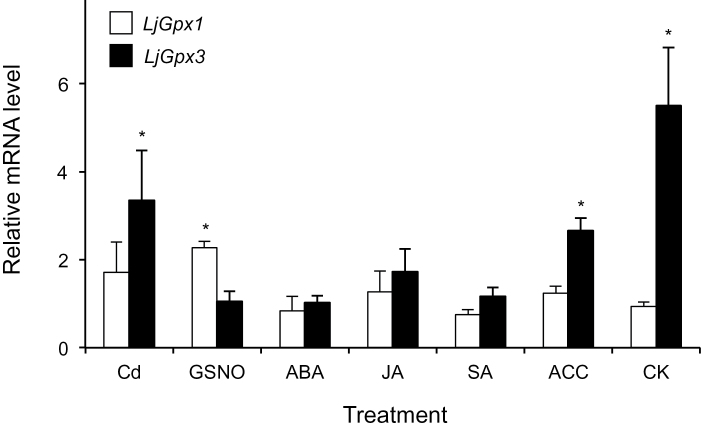
Expression of *LjGpx1* and *LjGpx3* in nodules of *L. japonicus* plants exposed to nitro-oxidative stress and hormones. Steady-state mRNA levels were normalized with respect to *ubiquitin* and are expressed relative to those of untreated plants, which were given an arbitrary value of 1. All data are means ± SE of 3–6 replicates. Asterisks denote significant up-regulation (>2-fold).

Firstly, the expression of *LjGpx1* and *LjGpx3* in nodules was analysed under nitro-oxidative stress elicited by cadmium (Cd), a heavy metal that promotes ROS production (Romero-Puertas *et al.*, 2004) or by GSNO, a NO-releasing metabolite implicated in *trans*-nitrosylation reactions (Perazzolli *et al.*, 2004). *LjGpx3* was upregulated by Cd whereas *LjGpx1* was responsive to GSNO (Fig. 2), which reflects differential gene regulation and strongly suggests an antioxidative role for the proteins. The effects of phytohormones at a physiologically relevant concentration (50 μM) on gene expression were also compared because at least some of them are mediated by NO (Bright *et al.*, 2006). *LjGpx3* but not *LjGpx1* was upregulated in nodules of plants treated with the ethylene precursor ACC or with CK, whereas the expression of the two genes was not affected by the stress signalling compounds ABA, JA, and SA (Fig. 2).

**Fig. 3.**
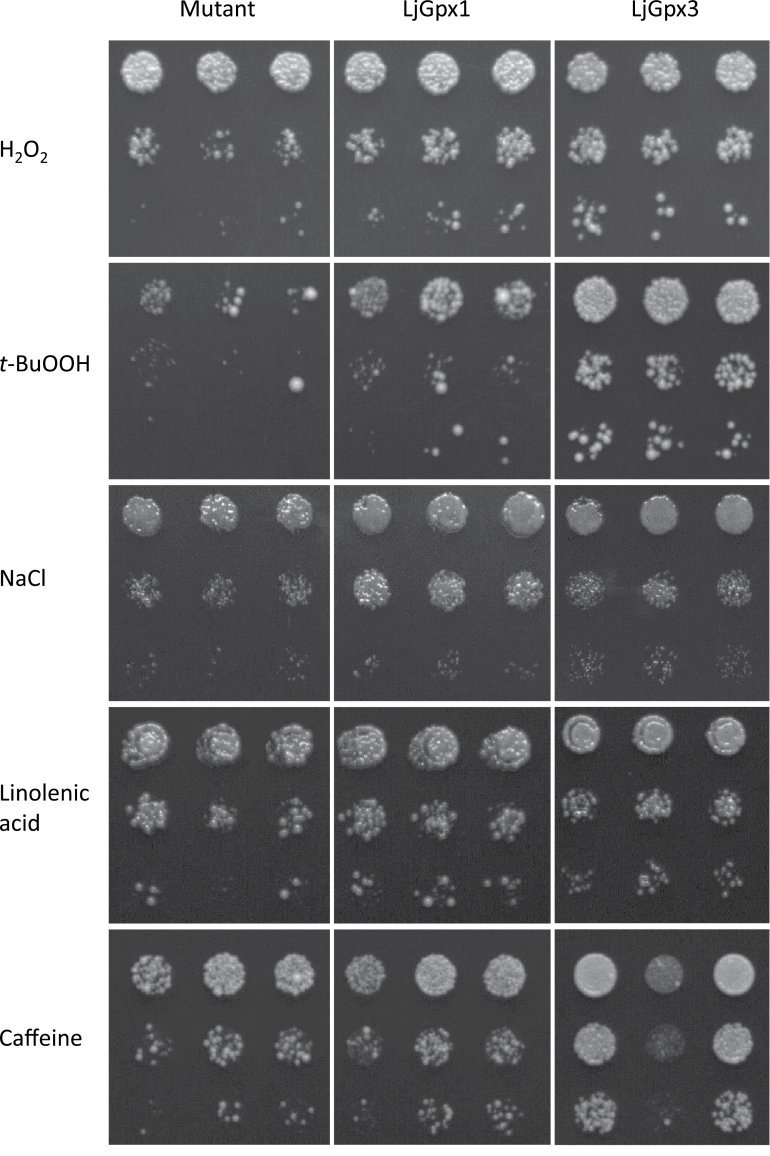
Functional complementation of LjGpx1 and LjGpx3 in yeast cells. The Gpx-deficient mutant and transformed cells were grown on YPD medium for 48h at 26°C with inducers of oxidative stress [500 μM H_2_O_2_ and 30 μM *t*-butyl hydroperoxide (*t*-BuOOH)], of salt stress (0.9M NaCl), and of membrane damage (1.5mM linolenic acid and 16mM caffeine). Serial dilutions (1:10, 1:100, and 1:1000) of saturated cultures (top to bottom), and three replicates (left to right), are shown on the plates. The whole experiment was repeated three times with similar results.

The second approach to assess the antioxidative role of LjGpxs was to perform functional complementation in yeast (Fig. 3). This strategy is extensively used with plant proteins, which in most cases are functional in yeast (Serrano *et al.*, 1999). Moreover, the use of yeast enabled us to examine directly the effects of LjGpx expression in a completely Gpx-null background. Thus, the effects of two peroxides, which are LjGpx substrates, on the growth of the yeast Gpx-deficient mutant and the corresponding transformed cells were investigated (Fig. 3). The concentrations of H_2_O_2_ and *t-*butyl hydroperoxide were optimized to maximize differences in phenotype. Both LjGpx1 and LjGpx3 complemented the defective growth of the mutant in the presence of H_2_O_2_ or *t-*butyl hydroperoxide (compare growth at the highest dilution, 1:1000). While the protection afforded by LjGpx1 and LjGpx3 against H_2_O_2_ was similar, LjGpx3 had a greater protective effect against *t-*butyl hydroperoxide. Likewise, yeast cells expressing LjGpx1 and LjGpx3 exhibited better growth than the mutant under salt stress induced by NaCl (Fig. 3). To determine whether LjGpx1 and LjGpx3 improve tolerance to stress imposed on the plasma membrane and/or the cell wall, yeast cells were treated with linolenic acid and caffeine. Yeast cells are unable to synthesize polyunsaturated fatty acids but incorporate exogenously added linolenic acid into the membranes, making them prone to peroxidation (Avery and Avery, 2001). Also, caffeine induces alteration of the yeast cell wall architecture and may affect membrane integrity (Kuranda *et al.*, 2006). Cells expressing either of the two LjGpx proteins showed greater tolerance to linolenic acid and caffeine than the mutant strain (Fig. 3).

To compare expression levels of LjGpx1 and LjGpx3 in yeast, antibodies were produced and used on immunoblots (Supplementary Fig. S2). Because the antibodies were intended to be employed also for immunolocalization studies of the LjGpxs, which require very high specificity, monospecific antibodies were purified from antisera by affinity chromatography and then repurified by immunoadsorption with *E. coli* protein extracts. This was necessary because very minor amounts of *E. coli* proteins inevitably contaminated the recombinant LjGpxs employed to raise the antibodies. The resulting antibodies specifically recognized recombinant LjGpx1 and LjGpx3 (Supplementary Fig. S2A) and the respective proteins of nodule extracts (Supplementary Fig. S2B). The use of these antibodies on immunoblots of yeast extracts revealed that LjGpx3 was expressed during the 48h of treatment with the peroxides and the other stress inducers, whereas LjGpx1 was only detectable at 12h and was probably degraded thereafter (Supplementary Fig. S2C). This may explain the higher tolerance to *t-*butyl hydroperoxide of yeast cells expressing LjGpx3 with respect to those expressing LjGpx1 (Fig. 3).

### LjGpx1 and LjGpx3 mRNAs are abundant in the nodule infected zone and the proteins are localized to various subcellular compartments

**Fig. 4.**
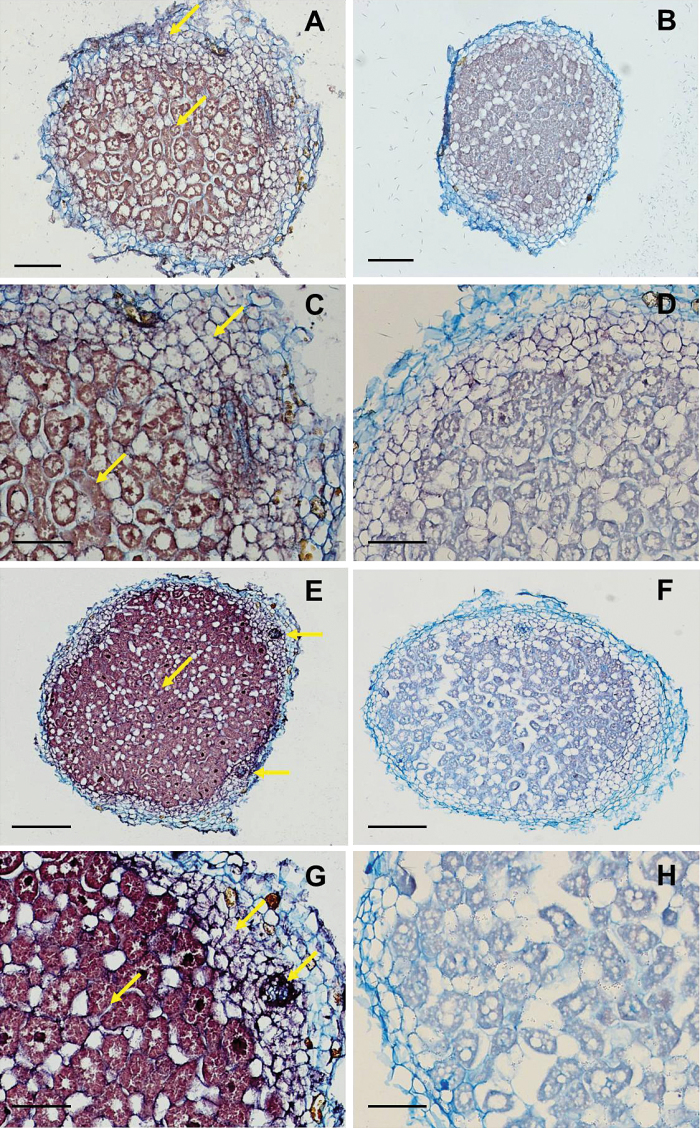
*In situ* mRNA hybridization of (A-D) *LjGpx1* and (E-H) *LjGpx3* in mature nodules (46-d-old plants). The figure shows nodule sections hybridized with antisense probes (A, C, E, and G) and with sense probes (B, D, F, and H) (negative controls). Arrows mark intense signal in the cortex, vascular bundles (for *LjGpx3*), and fixation zone. Bars, 75 μm (A, B, E, and F); 300 μm (C, D, G, and H).

*In situ* hybridization of mature nodules of *L. japonicus* was used to localize the mRNAs encoding the two LjGpx isoforms. The *LjGpx1* (Fig. 4A, C) and *LjGpx3* (Fig. 4E, G) mRNAs were found to be preferentially localized to the infected zone. Besides, significant amounts of *LjGpx3* mRNA could be detected in the nodule cortex and in the vascular bundles (Fig. 4E, G). However, in the case of *LjGpx1*, the control probe produced a signal in the cortex (Fig. 4B, D) and hence some non-specific signal in this nodule tissue cannot be ruled out. No background signal was seen for *LjGpx3*, confirming genuine expression of this gene in the nodule cortex (Fig. 4F, H).

**Fig. 5.**
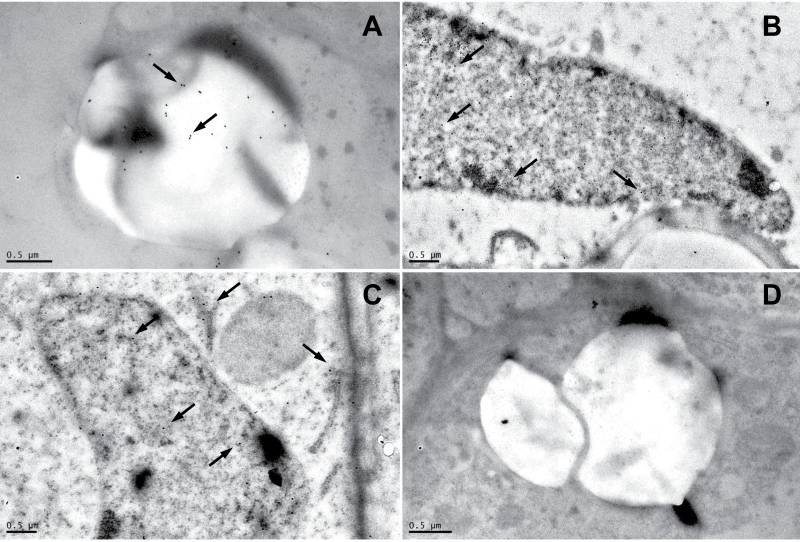
Immunogold localization of LjGpx1 and LjGpx3 in nodules. Micrographs show localization (arrows mark gold particles) of (A) LjGpx1 in amyloplast, (B) LjGpx1 in nucleus, and (C) LjGpx3 in endoplasmic reticulum, cytosol, and nucleus. (D) Negative control, in which non-immune serum substituted for antibodies against LjGpxs, shows the absence of labelling in amyloplast. Bars, 0.5 µm.

**Fig. 6.**
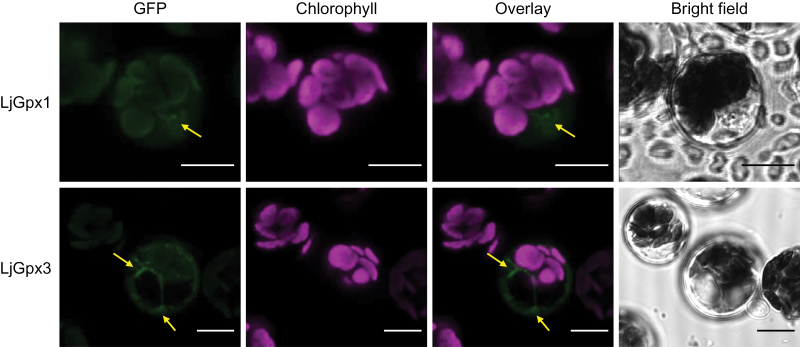
Subcellular localization of LjGpx1 and LjGpx3 using transient expression of GFP fusions in *A. thaliana* protoplasts. GFP fluorescence is depicted in green and chlorophyll autofluorescence in magenta. Arrows show localization of (A) LjGpx1 in nuclei and (B) LjGpx3 in the cytosol. Bars, 10 μm.

The LjGpx1 and LjGpx3 proteins were immunolocalized using our highly purified antibodies (Fig. 5). For LjGpx1, gold labelling was evident in the amyloplasts (Fig. 5A) and nuclei (Fig. 5B) of infected cells, cortical cells, and vascular bundle cells. For LjGpx3, gold particles were mainly associated to the endoplasmic reticulum, cytosol, and nuclei (Fig. 5C). A control in which preimmune serum replaced the primary antibodies did not show any labelling in the amyloplasts or nuclei (Fig. 5D). The immunolocalization study was complemented with fluorescence detection of the LjGpx-GFP fusion proteins by confocal microscopy. The constructs were transfected into *A. thaliana* mesophyll protoplasts (Fig. 6). GFP fluorescence was observed predominantly in the nuclei for LjGpx1 (Fig. 6A) and in the cytosol for LjGpx3 (Fig. 6B).

### LjGpx1 and LjGpx3 are nitrosylated *in vitro* and *in vivo*, which results in inhibition of enzyme activities

**Fig. 7.**
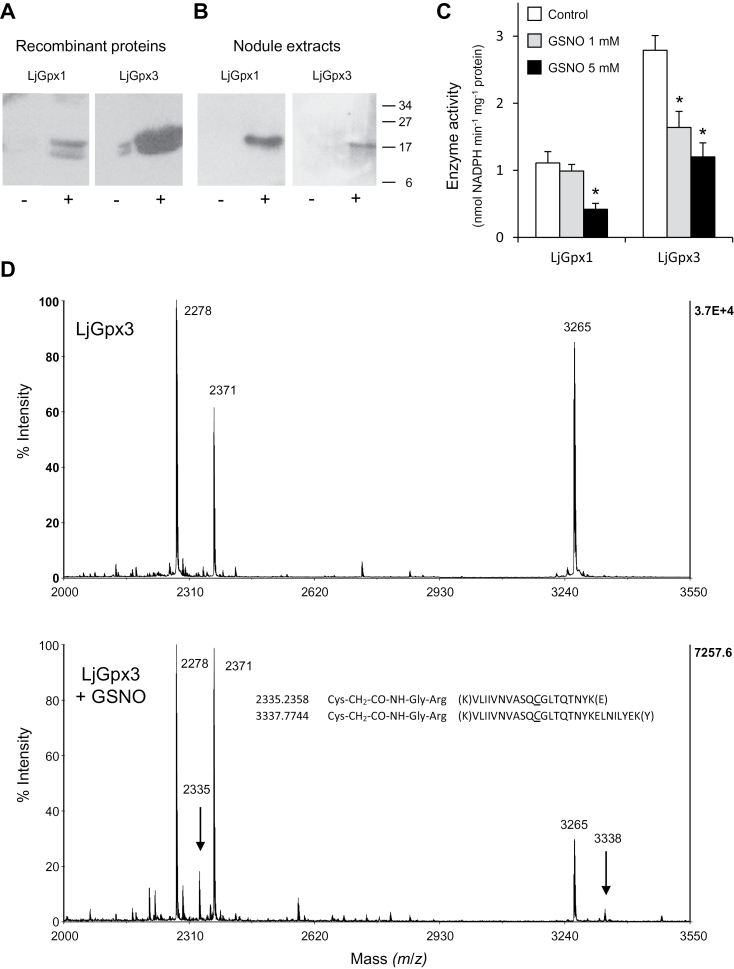
Nitrosylation of LjGpx1 and LjGpx3. (A) Immunoblot showing nitrosylation of purified LjGpx1 and LjGpx3. Recombinant proteins were treated with (-) 1mM GSSG (control) or with (+) 1mM GSNO, subjected to the biotin-switch, and immunoblotted with an anti-biotin antibody. (B) Immunoblot showing nitrosylation of LjGpx1 and LjGpx3 in nodule extracts from plants treated with 5mM GSSG (-) or with 5mM GSNO (+). Biotinylated proteins were affinity purified using streptavidin-agarose and immunoblotted with the LjGpx1 and LjGpx3 antibodies. (C) Effect of GSNO-mediated nitrosylation on LjGpx activities measured using the NADPH-coupled assay with poplar Trx*h1* and H_2_O_2_ as substrates. Recombinant LjGpx1 and LjGpx3 were treated with 1mM GSSG (control), 1mM GSNO, or 5mM GSNO for 1h at 37ºC. Values are means ± SE of 6–8 replicates. Means marked with an asterisk differ significantly from control at *P*<0.05 based on the Student’s *t*-test. (D) Mass spectra showing nitrosylation of Cys-85 using the His-tag switch. Esentially, the nitrosyl group of Cys is released by ascorbate and the free thiol is then alkylated by a synthetic peptide. During trypsinization, the synthetic peptide is cleaved, producing a Gly-Arg dipeptide that remains bound to the Cys via an amide bond. Arrows mark the presence of two peptides found in the tryptic digest of the nitrosylated protein (LjGpx3 + GSNO; *lower spectrum*), which are not present in the control unmodified protein (LjGpx3; *upper spectrum*). The molecular masses of these two peptides correspond to the alkylation of the Cys residue by the Gly-Arg dipeptide, as indicated in the figure.

Protein *S*-nitrosylation is an important mechanism by which NO exerts regulatory functions in all organisms (Astier *et al.*, 2012). To elucidate whether the biological activities of LjGpx1 and LjGpx3 could be modulated by NO, recombinant proteins were treated with 1mM GSNO and *S*-nitrosylation was evaluated with the biotin switch assay. Immunoblots showed that both proteins can be nitrosylated *in vitro* to some extent (Fig. 7A). Also, this treatment caused a 40% reduction of LjGpx3 activity but had no effect on LjGpx1 activity, whereas raising the GSNO concentration to 5mM resulted in a 60% loss of both LjGpx activities (Fig. 7C). Because the biotin switch does not permit the identification of nitrosylated Cys, the His-tag switch was used. This method involves derivatization of nitrosylated residues with a synthetic peptide. After trypsin digestion, the dipeptide Gly-Arg remains bound to Cys and can be detected by MS (Camerini *et al.*, 2007). The analysis demonstrated nitrosylation of Cys-85 in LjGpx3 (Fig. 7D), but could not prove the equivalent nitrosylation in LjGpx1 (see Supplementary Fig. S1 for numbering of Cys in the proteins).

In this analysis, a disulfide bond was detected between Cys-140 and Cys-159 in LjGpx1 and between Cys-114 and Cys-133 in LjGpx3. Addition of DTT before trypsinization to reduce the disulfide increased the peptide molecular mass by 2Da, confirming the existence of the intramolecular bond (Supplementary Fig. S3). Further controls were run by adding DTT to make all Cys accessible for nitrosylation and then removing it prior to the biotin switch. This result further proved that nitrosylation was restricted to Cys-85 of LjGpx3. The presence of disulfide bonds in LjGpx1 and LjGpx3 was already apparent on immunoblots of recombinant proteins or nodule extracts, in which two immunoreactive bands (reduced and oxidized forms) were seen for each protein (Fig. 7A).

Nodule extracts and intact plants were also incubated with 5mM GSNO for 6h and nodule proteins were subjected to the biotin switch assay. Biotinylation of Cys residues was observable after GSNO treatment of plants, but neither in nodule extracts nor in nodules of plants treated with GSSG (control), indicating that LjGpx1 and LjGpx3 are also amenable to nitrosylation *in vivo* (Fig. 7B).

## Discussion

Legume nodules are endowed with major antioxidant defences to keep ROS and NO under control, thus allowing the onset and functioning of symbiosis. In this work, LjGpx1 and LjGpx3, two isoforms abundantly expressed in nodules, were found to catalyse the efficient reduction of organic and lipid peroxides using Trx, but not GSH, as reductant (Table 1). Both enzymes are, therefore, Trx-dependent phospholipid hydroperoxidases, like other plant Gpxs for which kinetic parameters have been measured (Herbette *et al.*, 2002; Jung *et al.*, 2002; Navrot *et al.*, 2006). By using an affinity binding assay with a Cys-mutated Trx as a bait, it was shown that LjTrx*h4* forms intermolecular disulfide bonds with LjGpx1, LjGpx2, and LjGpx3 (Table 2), and hence that Trxs may act as *in vivo* reductants of LjGpxs. Although the specificity of the Trx isoform was not examined, LjGpxs may be targets of other LjTrxs because a high degree of interchangeability in the affinity column procedure was observed for poplar Trxs (Balmer *et al.,* 2003).

Further support for an *in vivo* role of LjGpxs as phospholipid hydroperoxidases is lent by complementation of a *S. cerevisiae gpx* mutant. This microorganism expresses three Gpxs, all of them identified as phospholipid hydro peroxidases (Avery and Avery, 2001). Accordingly, the triple-deletion mutant strain is hypersensitive to H_2_O_2_ and *t-*butyl hydroperoxide. Expression of LjGpx1 or LjGpx3 in the mutant yeast conferred greater tolerance to both peroxides (Fig. 3), indicating that the enzymes are functional. Furthermore, it indicates that LjGpxs successfully recruit endogenous Trxs as reductants. Because LjGpxs also afforded protection against linolenic acid, which sensitizes membranes to lipid peroxidation (Avery and Avery, 2001), and against caffeine, which also causes membrane lesions (Kuranda *et al.*, 2006), it is concluded that these enzymes protect against lipid peroxidation. Likewise, the beneficial effect of LjGpxs in yeast treated with high NaCl concentrations (Fig. 3) may be indirect and attributable to the Gpx capacity to offset oxidative stress, as proposed for the Gpx of salt-tolerant *Citrus sinensis* (Gueta-Dahan *et al.*, 1997; Avsian-Kretchmer *et al.*, 2004).

Because LjGpx1 and LjGpx3 show similar kinetic properties and are unlikely to be entirely redundant, the proteins may be differentially regulated by developmental and environmental cues, and/or be localized in different tissues, cells, or organelles. All this was found to be true. Thus, in nodules, *LjGpx1* was induced by NO and *LjGpx3* by Cd and some hormones (Fig. 2). Earlier work in our laboratory showed that *Gpx1* is down-regulated and *Gpx3* is up-regulated in roots of non-nodulated *L. corniculatus* plants treated with 20 μM Cd in hydroponic cultures (Ramos *et al.*, 2009). Although results are difficult to compare due to differences in plant species and tissues and in growth and treatment conditions, they show a consistent induction of the *Gpx3* gene with Cd in the two legumes. In *A. thaliana*, Gpx3 (At2g43350) is involved in the ABA response (Miao *et al.*, 2006) and expression of Gpx4 (At2g48150) and Gpx7 (At4g31870) is increased upon auxin application (Passaia *et al.*, 2014). In the present work, strong up-regulation of *LjGpx3* was seen with CK and less intense with ACC, but ABA had no effect. None of the tested hormones altered *LjGpx1* expression when applied at a concentration of 50 μM for 48h (Fig. 2). A direct comparison of results obtained with *A. thaliana* and *L. japonicus* is complicated because the functional equivalence of Gpx isoforms is uncertain (Rodriguez Milla *et al.,* 2003; Ramos *et al.*, 2009). However, based on the observations made with both model plants, it may be suggested that LjGpx1 and LjGpx3 isoforms have functions beyond antioxidative defence (see also discussion below). In particular, they might participate in signalling during plant development because their transcripts accumulated in response to hormones in healthy, non-stressed plants, which do not require an extra provision of antioxidants.

In a previous report, Gpx proteins were detected in root and nodule amyloplasts and in leaf chloroplasts of *L. japonicus* and other legumes (Ramos *et al.*, 2009) using an antibody raised against poplar Gpx3.2 (Navrot *et al.*, 2006). However, this antibody was not isoform specific and probably recognized several LjGpx proteins. In the present study, the tissue, cellular, and subcellular localizations of LjGpx1 and LjGpx3 were examined using mRNA *in situ* hybridization (Fig. 4), immunoelectron microscopy (Fig. 5), and fluorescence detection of GFP-tagged proteins (Fig. 6). The mRNA and protein levels of LjGpx1 and LjGpx3 are highest in the nodule infected zone. This pattern is in line with their requirement for antioxidative protection in the host cells, which contain copious amounts of symbiosomal membranes prone to peroxidation (Puppo *et al.*, 1991). Chloroplastic, cytosolic, and/or mitochondrial Gpxs have been reported in other vascular plants (Mullineaux *et al.*, 1998; Herbette *et al.*, 2004; Navrot *et al.*, 2006). *In silico* analyses predict that LjGpx1 bears a transit peptide for possible targeting to the plastids and mitochondria and that LjGpx3 is located to the cytosol and secretory pathway (Supplementary Fig. S1). For LjGpx1, immunogold labelling was detected in the nodule amyloplasts although GFP fluorescence was weak in *A. thaliana* chloroplasts. Neither technique supported the presence of LjGpx1 in mitochondria. In contrast, both of them indicated a nuclear localization. The immunolocalization study showed the presence of LjGpx3 in nuclei but this could not be confirmed by GFP tagging. Until now, only another plant Gpx, *A. thaliana* Gpx8 (At1g63460), was shown to be located to the nucleus (Gaber *et al.*, 2012). As for LjGpx3, immunogold labelling and GFP tagging were consistent with *in silico* analysis, indicating that the protein is in the cytosol and endoplasmic reticulum.

The differential regulation of *LjGpx1* and *LjGpx3* by the physiological NO donor GSNO and by phytohormones, along with the localization of LjGpx1 in the nuclei, provide indirect support for a role of LjGpxs beyond their antioxidant capacity. This possibility was tested by a more direct approach aimed at determining whether LjGpxs could be regulated by *S-*nitrosylation. The rationale for this set of experiments rests on the observations that LjGpxs contain Cys residues essential for catalytic activity (Supplementary Fig. S1) and that Cys nitrosylation is a major route for transmission of NO bioactivity (Astier *et al.*, 2012). In a first experiment, purified LjGpx1 and LjGpx3 were treated with GSNO and assayed for nitrosylation with the biotin switch (Fig. 7A). The nitrosylation of LjGpx3 was confirmed with the His-tag switch followed by MS and the target residue was identified as Cys-85 (Fig. 7D). However, LjGpx1 nitrosylation could not be verified probably because MS was performed with proteins treated with 1mM GSNO, a concentration at which LjGpx1 activity is not inhibited (Fig. 7C). Because the biotin switch is a reliable and sensitive method (Astier *et al.*, 2012), another likely explanation is that the equivalent Cys residue of LjGpx1 is not readily accessible to the peptide used for derivatization. In a second experiment, nodule extracts were treated with GSNO or were made from plants treated with GSNO, and were assayed with the biotin switch. The detection of nitrosylated LjGpxs indicates that both enzymes are targets of nitrosylation *in vitro* and *in vivo*. An intriguing question is why nitrosylation of the peroxidatic Cys (Cys-85) in LjGpx3 can take place while the resolving Cys (Cys-133) is present. Maybe the conformational changes normally occurring to bring the resolving Cys close to the peroxidatic Cys upon sulfenic acid formation cannot occur when Cys-85 is nitrosylated. The fact that nitrosylation of the peroxidatic Cys inhibits enzyme activity, even in the presence of the Trx-reducing system, probably reflects the inability of Trx to readily reduce the Cys-NO adduct. The MS analysis also pointed out the formation of a disulfide bond between the second and third Cys in both LjGpxs (Supplementary Fig. S3), as reported for a Chinese cabbage (*Brassica rapa*) Gpx (Jung *et al.*, 2002). This disulfide may regulate enzyme activity as it entails the third (resolving) Cys, required for Trx-mediated regeneration (Navrot *et al.*, 2006; Koh *et al.*, 2007). None of the two other possible internal disulfide bonds was detected. In particular, the disulfide bond between the first and third Cys, essential for enzyme activity (Koh *et al.*, 2007), may have been missed because it involves two different tryptic peptides, which is often recalcitrant to MS analysis. Overall, the observed GSNO-dependent inhibition of Gpxs may contribute to the transient increase of the concentration of their targeted substrates, such as lipid hydroperoxides, thus interconnecting NO and ROS signalling pathways, which are known, for example, to play complementary roles during the plant immune response (Zaninotto *et al.*, 2006).

In summary, an extensive study of two Gpx isoforms abundant in legume nodules has been conducted. LjGpx1 and LjGpx3 are phospholipid hydroperoxidases capable of interacting *in vitro* with Trxs endogenously present in nodules such as Trx*h4*. The enzymes protect from oxidative stress and membrane damage, are highly expressed in the nodule infected cells, and are located to different cellular compartments. At least the LjGpx1 isoform is present in the nucleus. Differential expression of LjGpx1 and LjGpx3 in response to GSNO and hormones, localization in nuclei, and susceptibility to nitrosylation of the catalytic Cys *in vitro* and probably *in vivo* provide strong support for signalling roles in addition to their antioxidative properties.

## Supplementary data

Supplementary data are available at *JXB* online

Supplementary Fig. S1. Amino acid sequence alignment of representative Gpxs mentioned in this work.

Supplementary Fig. S2. Immunoblots showing the specificity of the LjGpx1 and LjGpx3 antibodies, and the expression of both proteins in nodules and in transformed yeast cells.

Supplementary Fig. S3. MS analysis demonstrating the presence of a disulfide bond between Cys-114 and Cys-133 in LjGpx3.

Supplementary Table S1. Oligonucleotides used in this study.

Supplementary Data

## Abbreviations:

ABA: abscisic acid
ACC: 1-aminocyclopropane-1-carboxylic acid
CK: cytokinin
Gpx: glutathione peroxidase
GSH: glutathione
GSNO: *S*-nitrosoglutathione
GSSG: glutathione disulfide
JA: jasmonic acid
NO: nitric oxide
ROS: reactive oxygen species
SA: salicylic acid
Trx: thioredoxin.
