# Supplementary material for: Function of glutathione peroxidases in legume root nodules
Source: J Exp Bot. 2015 Mar 4;66(10):2979–90. doi: 10.1093/jxb/erv066 (PMC4423513; doi:10.1093/jxb/erv066)
Supplement: Supplementary Data [file supp_66_10_2979__index.html]

Function of glutathione peroxidases in legume root nodules — Function of glutathione peroxidases in legume root nodules — Supplementary Data 

# Function of glutathione peroxidases in legume root nodules

## Supplementary Data

Data files

**Files in this Data Supplement:**

- Supplementary Data - Supplementary Data
