# Supplementary material for: Function of glutathione peroxidases in legume root nodules
Source: J Exp Bot. 2015 Mar 4;66(10):2979–90. doi: 10.1093/jxb/erv066 (PMC4423513; doi:10.1093/jxb/erv066)
Supplement: Supplementary Data [file supp_erv066_jexbot144261_file001.pdf]

Table S1. *Oligonucleotides used in this study*

fw, forward primer (5' → 3'); rv, reverse primer (5' → 3'). Reference genes: *LjUBQ*, ubiquitin; *LjeIF-4A*, eukaryotic initiation factor 4A; *LjPP2A*, protein phosphatase 2A.

**Production of recombinant proteins**	
LjGpx1 (fw)	CAC CAT GGC TGC CCC CAC AT
LjGpx1 (rv)	TCA TGC ACC CAA CAA TTT CAC
LjGpx3 (fw)	CAC CAT GGC TGA ACA AAC CT
LjGpx3 (rv)	TCA AGA AGA TTG TAA GAG CT
LjTrxh4 (fw)	CAC CAT GGG CGG AGT CCT CTC T
LjTrxh4 (rv)	CTA AGC TCG GAG CTG CTC A
**Site-directed mutagenesis**	
LjTrxh4 (fw)	TGG TGC GGG CCG AGC CGG TTC ATA G
LjTrxh4 (rv)	CTA TGA ACC GGC TCG GCC CGC ACC A
***In situ* hybridization probes**	
LjGpx1 (fw)	TCT GTA CTC GCA TCT TGT TCT TCT
LjGpx1 (rv)	TGA TCT GGT CTG AGA GTG AAA GAG
LjGpx3 (fw)	AGT TGT TGA CTT TCT GGA ATT GG
LjGpx3 (rv)	ACT CAC ATC ATT TCC ACG GAT
LjGpx1 T7 (fw)	ATT ATG CTG AGT GAT ATC CCT CTG TAC TCG CAT CTT GTT CTT CT
LjGpx1 T7 (rv)	ATT ATG CTG AGT GAT ATC CCT GAT CTG GTC TGA GAG TGA AAG AG
LjGpx3 T7 (fw)	ATT ATG CTG AGT GAT ATC CCA GTT GTT GAC TTT CTG GAA TTG G
LjGpx3 T7 (rv)	ATT ATG CTG AGT GAT ATC CCA CTC ACA TCA TTT CCA CGG AT
**Quantitative reverse-transcription PCR**	
LjGpx1 (fw)	ACT ATG GCT GCC CCC ACA T
LjGpx1 (rv)	TCT GGC ATC TTT GAC GGT GA
LjGpx3 (fw)	TGG GAA GAA TGC AGA ACC ACT
LjGpx3 (rv)	CCC CCT TTC TGA TCC TTC AAA
LjGpx1 (fw)	ACT ATG GCT GCC CCC ACA T
LjUBQ (fw)	TTC ACC TTG TGC TCC GTC TTC
LjUBQ (rv)	AAC AAC AGC ACA CAC AGA CAA
LjeIF-4A (fw)	AGA GGG TTT AAA GAT CAA AT
LjeIF-4A (rv)	ATG TCA ATT CAT CAC GTT TT
LjPP2A (fw)	TGA GCT ATG TGA AGC TGT TGG T
LjPP2A (rv)	CAG CCT CAT TAT CAC GCA GTA G

```
PtGpx3      MLTSRSRILSQKYLNFASLSASFLLSKQSSFNSKQTLLPSLHNSPVSLYSQSIKAGVSRR
AtGpx6             MLRSSIRLLYIRRTSPLLRSLSSSSSSSSSKRFDSAKPLFNSHRIISLPISTT
LjGpx1       MLCTRILFFSRTIRFAAPLSSSSLHSFVFSNSPITLSRSYHSSLLTTTSFPIKSLVSTS
LjGpx3                                 MQLLTFWNWISLVILAFAFFFFFFFFFFFFYSQ
AtGpx3                                      MPRSSRWVNQRATSKIKKFILFLGVAFV
BrGpx            MASSSYAPFSAVFSGFAATKPNPPPTCSAFLVPKRRSNSRNLKNGVSLKSWNKHG

                                                           *
PtGpx3      LLGSVRFNHSMASQSSPQSAHDFTVKDAKGNDVDLSIYKGKVLLIVNVASQCGLTDSNYT
AtGpx6      GAKLSRSEHSMAASSEPKSLYDFTVKDAKGNDVDLSIYKGKVLLIVNVASQCGLTNSNYT
LjGpx1      TTPFSFTLRPDHTMAAPTSVYDFTVKDARGNDVNLGDYKGKVLLIVNVASQCGLTNSNYT
LjGpx3      THPASPPSPSTMAEQTSKSLYDFTVKDIRGNDVSLSQYSGKVLIIVNVASQCGLTQTNYK
AtGpx3      FYLYRYPSSPSTVEQSSTSIYNISVKDIEGKDVSLSKFTGKVLLIVNVASKCGLTHGNYK
BrGpx       FQFTSRNLSVYARATEEKTVHDFTVKDISGKDVSLDKFKGKPLLIVNVASKCGLTSSNYT

                                   *
PtGpx3      ELTQLYAKYKDQGLEILAFPCNQFGSQEPGSSEEIVEFACTRFKAEYPIFDKVEVNGNNA
AtGpx6      ELAQLYEKYKGHGFEILAFPCNQFGNQEPGTNEEIVQFACTRFKAEYPIFDKVDVNGDKA
LjGpx1      ELSQLYEKYKSKGLEILGFPCNQFGAQEPGDNEQIQEFVCTRFKAEFPVFDKVDVNGDSA
LjGpx3      ELNILYEKYKSKGLEILAFPCNQFAGQEPGTNDEIQDVVCTRFKSEFPVFDKVEVNGKNA
AtGpx3      EMNILYAKYKTQGFEILAFPCNQFGSQEPGSNMEIKETVCNIFKAEFPIFDKIEVNGKNT
BrGpx       ELSQLYDKYRNQGFEILAFPCNQFGGQEPESNPDIKRFVCTRFKAEFPIFDKVDVNGPST

                                 *
PtGpx3      APIYKYLKSSKGGLFGDNIKWNFSKFLVDKEGKVVDRYAPTTSPLSIEKEVKKLLGIA
AtGpx6      APVYKFLKSSKGGLFGDGIKWNFAKFLVDKDGNVVDRFAPTTSPLSIEKDVKKLLGVTA
LjGpx1      APLYKYLKSSKGGLFGDKIKWNFSKFLVDKEGNVVERYAPTTSPLSIEKDLVKLLGA
LjGpx3      EPLFKFLKDQKGGIFGDGIKWNFTKFLVNKEGKVVERYAPTTSPMKIEKDLEKLLQSS
AtGpx3      CPLYNFLKEQKGGLFGDAIKWNFAKFLVDRQGNVVDRYAPTTSPLEIEKDIVKLLASA
BrGpx       APIYQFLKSKSGGFLGDLIKWNFEKFLVDKKGNVVQRYPPTTSPLQIEKDIQKLLVA
```

Fig. S1. Amino acid sequences of representative Gpxs mentioned in this work. The three Cys residues are indicated in red, the catalytic triad is marked with an asterisk, the conserved domains are highlighted in yellow, and the putative transit peptides of LjGpxs are highlighted in blue. At, *Arabidopsis thaliana* (Rodriguez Milla *et al.*, 2003); Br, *Brassica rapa* (Jung *et al.*, 2002); Lj, *Lotus japonicus* (Ramos *et al.*, 2009); Pt, *Populus trichocarpa* (Navrot *et al.*, 2006).

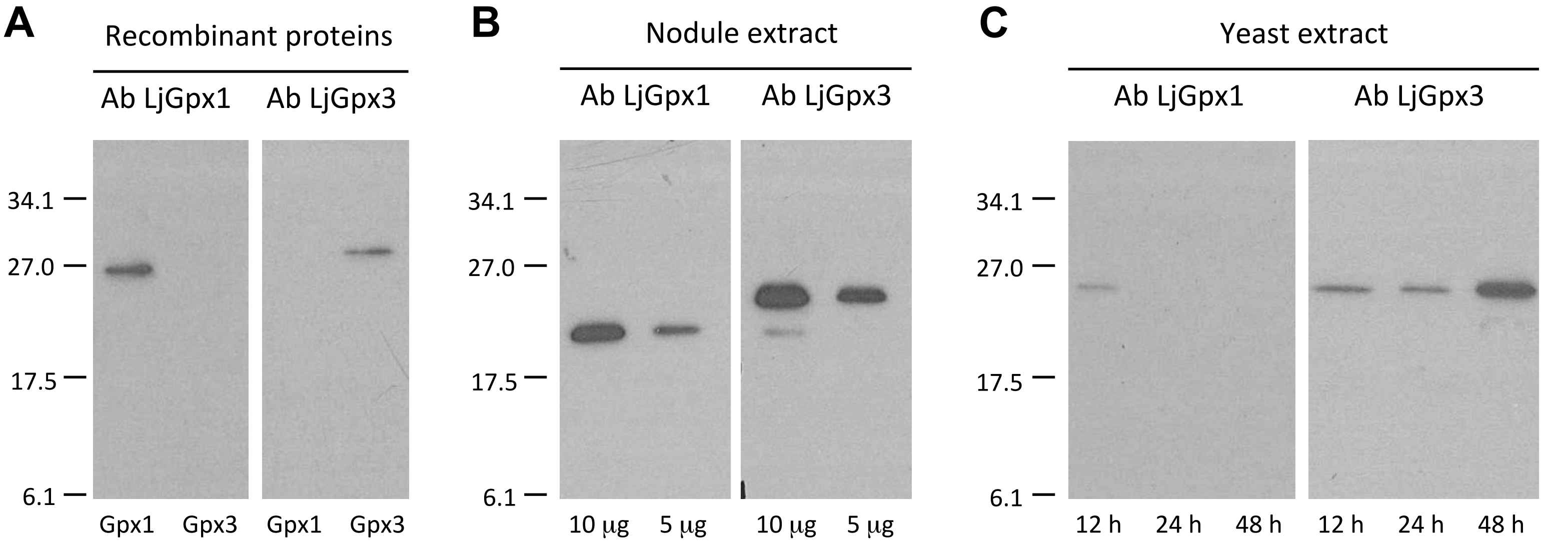

Fig. S2. Immunoblots showing the specificity of the LjGpx1 and LjGpx3 antibodies, and the expression of the two proteins in nodules and in transformed yeast cells. (A) Antibodies (Abs) recognize the respective purified recombinant proteins LjGpx1 and LjGpx3 (1 ng protein per lane). (B) Abs recognize the proteins in nodule extracts (5 or 10 μg protein per lane). (C) Abs recognize the proteins in transformed yeast cells (40 μg protein per lane). Note that LjGpx3 was visible after 12, 24, and 48 h of cell growth, whereas LjGpx1 was only visible at 12 h but not afterwards.

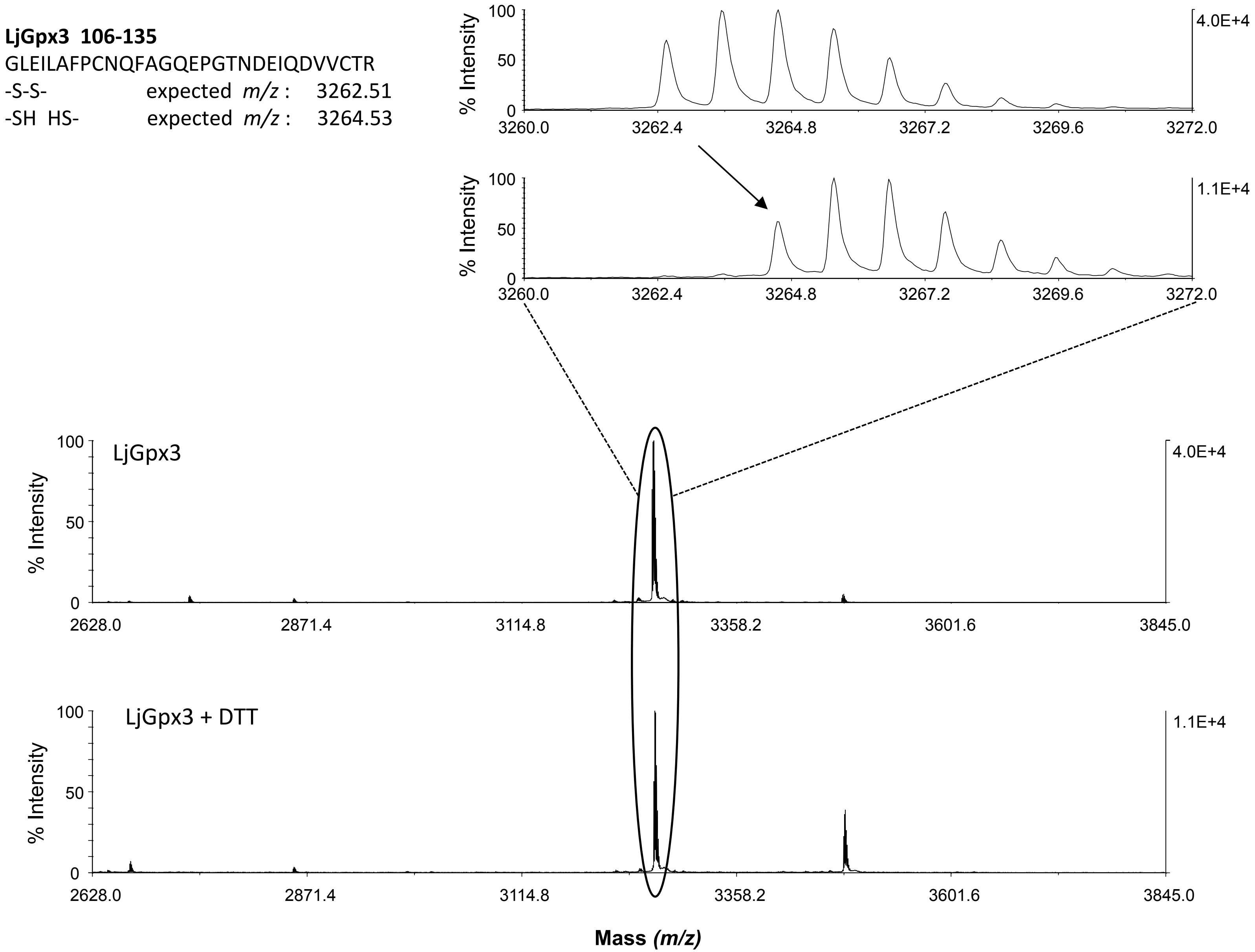

Fig. S3. MS analysis demonstrating the presence of a disulfide bond between Cys-114 and Cys-133 in LjGpx3. The molecular mass of the relevant peptide from control LjGpx3 (*m/z* 3262.51) was shifted 2 mass units (*m/z* 3264.53), corresponding to the 2 H of the two thiol groups, when the protein was incubated with DTT prior to trypsinization. Identical MS method was followed to demonstrate the presence of a disulfide bond between the equivalent Cys residues (Cys-140 and Cys-159) in LjGpx1.
